# Comparative transcript profiling of the fertile and sterile flower buds of *pol* CMS in *B. napus*

**DOI:** 10.1186/1471-2164-15-258

**Published:** 2014-04-03

**Authors:** Hong An, Zonghui Yang, Bin Yi, Jing Wen, Jinxiong Shen, Jinxing Tu, Chaozhi Ma, Tingdong Fu

**Affiliations:** 1 National Key Lab of Crop Geneticc Improvement, National Center of Crop Molecular Breeding Technology, National Center of Oil Crop Improvement (Wuhan), College of Plant Science and Technology, Huazhong Agricultural University, Wuhan 430070, P. R. China

**Keywords:** *Brassica napus*, *pol* CMS, Anther, Transcriptome

## Abstract

**Background:**

The Polima (*pol*) system of cytoplasmic male sterility (CMS) and its fertility restoration gene *Rfp* have been used in hybrid breeding in *Brassica napus*, which has greatly improved the yield of rapeseed. However, the mechanism of the male sterility transition in *pol* CMS remains to be determined.

**Results:**

To investigate the transcriptome during the male sterility transition in *pol* CMS, a near-isogenic line (NIL) of *pol* CMS was constructed. The phenotypic features and sterility stage were confirmed by anatomical analysis. Subsequently, we compared the genomic expression profiles of fertile and sterile young flower buds by RNA-Seq. A total of 105,481,136 sequences were successfully obtained. These reads were assembled into 112,770 unigenes, which composed the transcriptome of the bud. Among these unigenes, 72,408 (64.21%) were annotated using public protein databases and classified into functional clusters. In addition, we investigated the changes in expression of the fertile and sterile buds; the RNA-seq data showed 1,148 unigenes had significantly different expression and they were mainly distributed in metabolic and protein synthesis pathways. Additionally, some unigenes controlling anther development were dramatically down-regulated in sterile buds.

**Conclusions:**

These results suggested that an energy deficiency caused by *orf224/atp6* may inhibit a series of genes that regulate pollen development through nuclear-mitochondrial interaction. This results in the sterility of *pol* CMS by leading to the failure of sporogenous cell differentiation. This study may provide assistance for detailed molecular analysis and a better understanding of *pol* CMS in *B. napus.*

## Background

A very important source of vegetable oil worldwide, *Brassica napus* has extremely high oil production efficiency [1], and *pol* cytoplasm has been widely used in most of cultivated breeds [2]. Cytoplasmic male sterility (CMS) prevents self-pollination through pollen abortion, enabling the use of heterosis in hybrid crops for genetic improvement [3,4]. The sterility mechanism of *pol* has been examined in many studies, in which the *orf224/atp6* and *Rfp* genes have mainly been analyzed. Results have shown that the sterility is caused by chimeric mitochondrial genes regulated by nuclear genes [2,5-8].

In recent years, high-throughput sequencing methods such as Illumina SOLEXA, ABI SOLiD and Roche 454 have observably increased the efficiency and reduced the cost of sequencing, making the study of transcriptomes and even genome levels easier and more feasible [9]. Nowadays, the transcriptomes of many higher plants have been sequenced for different purposes, including *Arabidopsis thaliana*[10,11], *Myrica rubra*[12], *Fagopyrum*[13], *Citrus sinensis*[14], *Carthamus tinctorius*[15], and even *Brassica napus* for fertility studies [16]. By RNA-Seq, researchers can obtain almost all of the expressed genes, especially genes with very low abundance. Therefore, genes with abundant expression differences and interesting pathways can be analyzed exhaustively [17]. Additionally, RNA-Seq also has great advantages in the identification of new genes and SNPs, and even in genome-wide association studies (GWAS) [18-20]. In polyploid plants, it is used to study the fate of duplicated genes as well, such as in soybean and bread wheat [21,22].

In this work, fertile and sterile flower buds of *pol* CMS with a length of 0–1 mm were sequenced using the Illumina high-throughput sequencing platform, representing the first study of the *pol* CMS genome at the transcriptome level. The aim of this work was to identify the differences between the fertile and sterile buds at the transcriptional level, and find out the different bioprocesses involved and their related functions. These results will help the elucidation of the sterility molecular mechanism, and assist the breeding of *B. napus*.

## Results

### Phenotypic characterization of fertile and sterile floral buds

**Figure 1:**
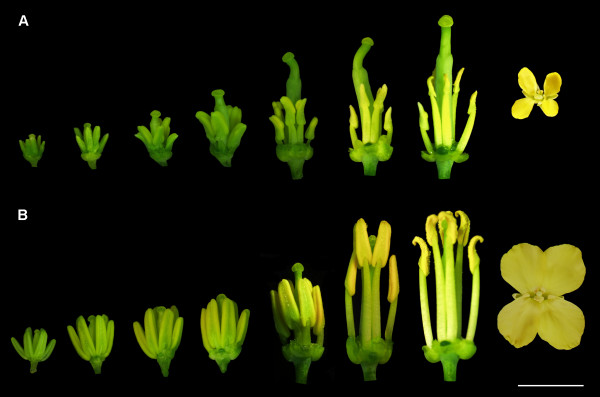
**Phenotypic characterization of fertile and sterile floral buds. A**: phenotype of sterile floral buds; **B**: phenotype of fertile floral buds. Bar for front-view is 5 mm, and for vertical-view is 15 mm.

**Figure 2:**
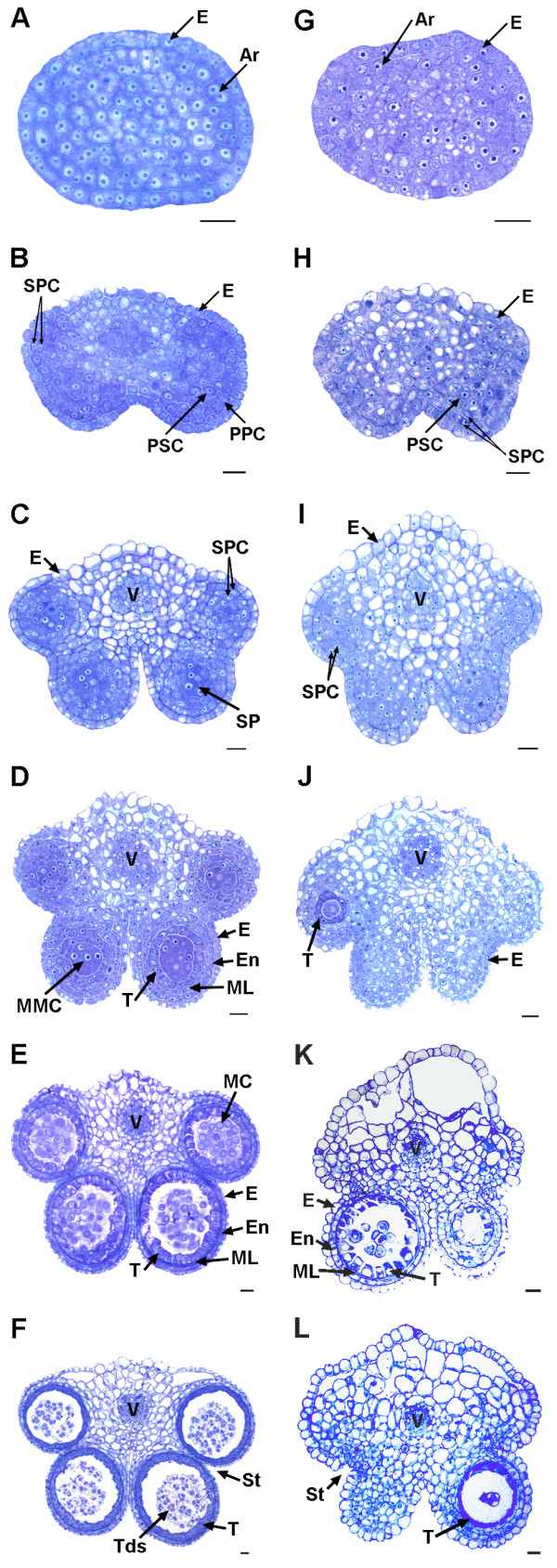
**Development of fertile (A-F) and sterile (G-L) anthers in *pol *CMS.** Bar = 10 μm for all the stages. Ar, archesporial cell; E, epidermis; En, endothecium; MC, meiotic cell; ML, middle layer; MMC, microspore mother cells; PPC, primary parietal cell; PSC, primary sporogenous cell; SP, sporogenous cell; SPC, secondary parietal cell; St, stomium; T, tapetum; Tds, tetrads; V, vascular region. **A** to **F** represent the anther development stage 2 to 7, respectively. So do G to L [25].

Sterile flowers were visually smaller than fertile flowers. In the developmental process, the anthers and filaments of the sterile flowers were always shorter than those of the fertile flowers. In addition, the sterile anthers produced little or no pollen, but the pistil was normal (Figure 1). To accurately determine the stage and tissue in which sterility occurs, fertile and sterile anthers were cytologically observed in different development stages. As shown in Figure 2, there was no clear difference between them until stage 4 of anther development. In stage 4, the sterile anthers did not differentiate sporogenous cells; additionally, the middle layer, endothecium and tapetum could not be distinguished (Figure 2C,I). After that, the sterile anthers were filled with numerous highly vacuolated cells. They also showed cavities in later stages (Figure 2K,L). In sterile anthers, locules with pollen sacs in stage 4 were very few and did not produce normal tetrads. In contrast, the fertile anthers developed normally in all stages.

### Transcriptome sequencing and assembly

After the raw data were trimmed, 62,417,937 clean reads for fertile samples and 43,063,199 for sterile samples were obtained (Additional file 1). All clean reads (105,481,136) were assembled by running Trinity, as no reference genome was available for *B. napus*[23]. As a result, 164,874 contigs (> 200 bp) were generated with an average length of 621 bp and a N50 of 927 bp. After clustering, 112,770 unigenes were obtained, including 22,306 clusters and 90,464 singletons. The average length of these unigenes was 638 bp and the N50 was 982 bp. There were 70,956 unigenes (62.92%) with a length range from 200 to 500 bp, 20,178 unigenes (17.89%) longer than 1,000 bp and no unigenes shorter than 200 bp (Table 1). The singletons mentioned above refer to contigs that matched no other contig, and the clusters and singletons generated from the contigs covered all of the obtained unigenes [24].

**Table 1:** **Summary of *de novo *** **transcriptome**

**Length (bp)**	**Contigs**	**Percentage (%)**	**Unigenes**	**Percentage (%)**
**200-499**	105,020	63.70	70,956	62.92
**500-999**	32,032	19.43	21,636	19.19
**1000-1499**	13,678	8.30	9,677	8.58
**1500-1999**	7,304	4.43	5,404	4.79
**> = 2000**	5,840	3.54	5,097	4.52

### Functional annotation

**Figure 3:**
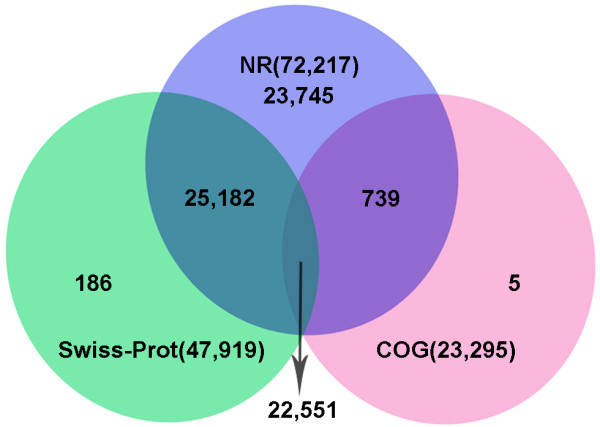
**Unigenes annotated with public databases.** The numbers of annotated unigenes were signified in the different regions.

For annotation, the 112,770 unigenes were subjected to BLASTX searches against the sequences in the NR (non-redundant protein sequences in NCBI), Swiss-Prot and COG databases (E-value ≤ 1e-5). As a result, 72,217 (64.04%), 47,919 (42.49%) and 23,295 (20.66%) unigenes were aligned against the three protein databases, respectively. Among these unigenes, 22,551 (20.00%) were annotated by all three databases and 72,408 (64.21%) were annotated by at least one database (Figure 3).

**Figure 4:**
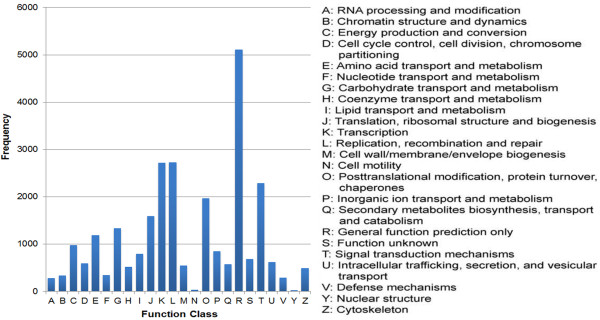
**COG function classification.** All the unigenes aligned in COG database were assorted in 24 clusters.

**Figure 5:**
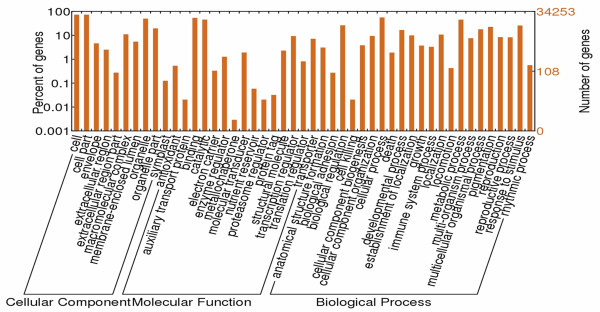
**Classification of GO annotations.** The x-axis indicates the sub-categories; the left y-axis indicates the percentage of a sub-category of genes in that category and the right y-axis indicates the number of unigenes in a sub-category.

There were 23,290 unigenes annotated by both the NR and COG databases. To examine the distribution of the assembly results, these unigenes were categorized into at least 24 COG (Clusters of Orthologous Groups) function clusters. Among the 24 clusters, the “general function prediction only” cluster comprised the highest number of unigenes (5,113, 21.95%), and the “replication, recombination and repair” cluster had the second largest number of unigenes. In contrast, only 23 unigenes were classified into “nuclear structure” (Figure 4). GO (Gene ontology) classifications were also obtained to investigate the functions of the unigenes; 47,733 (42.33%) unigenes that were annotated by both the NR and Swiss-Prot databases were classified into 47 groups. Among the 47,733 unigenes, 34,252 were assigned 158,341 GO annotations. All 47 groups can be categorized into three main classifications: “cellular component”, “molecular function” and “biological process”. There were 24,928 (72.78%) unigenes in the “cell part”, 18,352 (53.58%) in the “binding” and 19,118 (55.82%) in the “cellular process” categories, which were the major categories in each of the three main classifications motioned above, respectively. In addition, “organelle”, “catalytic activity” and “metabolic process” also had large proportions (> 45%) of unigenes. Conversely, some groups such as “metallochaperone activity”, “cell killing”, “proteasome regulator activity” and “auxiliary transport protein activity” had small numbers of unigenes (Figure 5).

### Putative genes related with pollen development

The development of pollen is a fundamental and complex process in flowering plants. It is essential for propagation and evolution. As a model plant, *Arabidopsis thaliana* has been well studied for this bioprocess. Therefore, all of the unigenes identified here were annotated to the TAIR database (http://www.arabidopsis.org/). Subsequently, unigenes annotated to 19 genes from *AG* to *MS1*, which are considered to regulate pollen development and act before meiosis, were extracted for analysis [25-29]. The results showed that among the 19 genes, some were significantly down-regulated in sterile buds, such as *BnNZZ/SPL, BnSPL8*, *BnEXS/EMS1* and *BnER*, but no gene was dramatically up-regulated (Additional file 2). Additionally, most of these genes did not show significant differences in expression between the fertile and sterile buds.

### Transcripts differentially expressed in fertile and sterile buds

The FPKM (Fragments per Kilobase of transcript per Million mapped reads) value was calculated to test the expression levels of the unigenes. We noticed that in some basic bioprocesses these unigenes showed high expression levels in both the fertile and sterile buds. For example, unigene78635 and unigene78636, which were involved in RNA transport, showed very high expression in both fertile buds (FPKM = 14,059.7 and FPKM = 9,451.37) and sterile buds (FPKM = 12,954.3 and FPKM = 8,286.57). Additionally, some unigenes related to metabolism, such as unigene10390 (FPKM > 4,300), showed high abundance as well.

**Figure 6:**
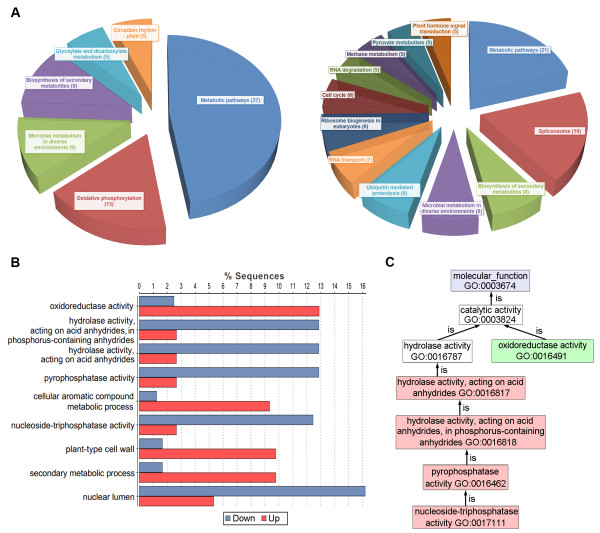
**Comparison of annotations between up- and down-regulated unigenes in sterile buds. A**: a pie chart of KEGG annotation in up-(left) and down-(right) regulated unigenes (n > 4), respectively; **B**: a statistical column diagram of GO annotation; **C**: an enriched graph of molecular function in GO annotation. They were filtered using default value.

With the restrictive conditions of log_2_ ratio > 1.0 and P-value < 0.001, a total of 1,148 (1.02% of all unigenes) unigenes that were significantly differentially expressed were obtained, including 629 up-regulated and 519 down-regulated unigenes in sterile buds (Additional file 3). The up- and down-regulated unigenes were submitted to KAAS (KEGG Automatics Annotation Server) and classified with the SBH (single directional best hit) method [30]. Most of the up-regulated unigenes were included in metabolic pathways (Figure 6A). In contrast, a relatively large number of the down-regulated unigenes functioned in transcription and translation besides metabolic pathways (Figure 6A), such as the unigenes related with spliceosomes, RNA transport and ribosome biogenesis. Specifically, we found that there were seven callose synthase (K11000) genes in the down-regulated unigenes. This result was consistent with the observation of anther semi-thin sections.

In addition, GO analysis was performed using Blast2GO [31]. After enrichment analysis, only 9 GO terms showed significant differences. Among these terms, four (“oxidoreductase activity”, “cellular aromatic compound metabolic process”, “secondary metabolic process” and “cell wall”) were associated with the up-regulated unigenes and five (mainly hydrolase, pyrophosphatase and nucleoside-triphosphatase activity, and “nuclear lumen”) were associated with down-regulated unigenes (Figure 6B). In the molecular function category, only oxidoreductase and hydrolase activities were different between the up- and down-regulated unigenes (Figure 6C).

In particular, *orf224*, which is believed to be a key ORF of *pol* CMS in the mitochondrial genome [5], showed a 4-fold transcript increase in sterile buds compared with fertile buds. *Atp6*, a chimeric gene of *orf224*, also showed minor up-regulation.

### qRT-PCR verification

**Figure 7:**
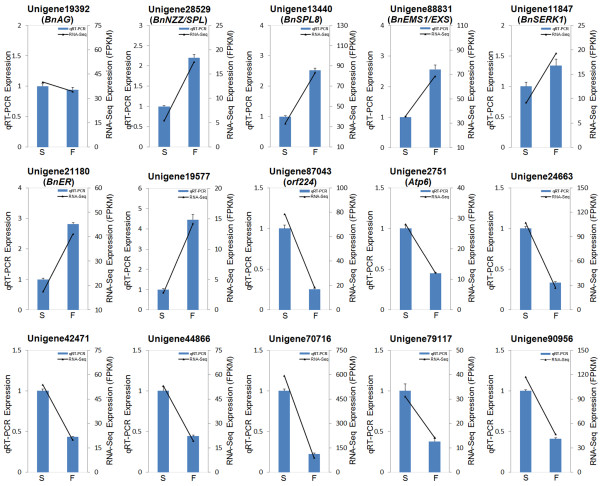
**qRT-PCR verification of differentially expressed unigenes.** S means sterile sample and F means fertile sample.

The differentially expressed unigenes were selected to verify the accuracy of the RNA-Seq analysis by qRT-PCR, using the same RNA that was used for RNA-Seq. Even though most qRT-PCR results indicated smaller differences compared with the RNA-Seq analysis, there was a consistent expression tendency (Figure 7). However, unigene3022 was an exception. In qRT-PCR, unigene3022 showed no difference in the fertile and sterile buds, while RNA-Seq analysis indicated a significant difference. qRT-PCR verified that among the five unigenes annotated to pollen development, four (unigene28529, unigene13440, unigene88831, unigene21180) showed low abundance in sterile buds and one (unigene19392, which was the earliest expressed) showed no difference between fertile and sterile buds (Figure 7). Unigene19577 and unigene2959, which were predicted to encode callose synthases, showed down-regulation in sterile buds. Unigene87043 and unigene2751, mitochondrial genes that influence fertility directly, were confirmed to be up-regulated in sterile buds. In addition, unigene44866, unigene24663 and unigene42471, which participate in the glycolysis/gluconeogenesis pathway, amino acid metabolism and oxidative phosphorylation, respectively, were all up-regulated in sterile buds.

## Discussion

Here, the transcriptome reads of fertile and sterile buds were acquired using the Illumina sequencing platform. Then, because of the unavailability of the genome and transcriptome of *B. napus* at present, we assembled the transcriptome of the buds for further research. Altogether, 112,770 unigenes were obtained, but only 1,148 unigenes (1.02% of all unigenes) showed notable differences in expression, indicating that though the development of buds is a complicated and polygenic process, changes in a relatively small number of genes can transform the trait observably.

In the present study, unigenes that were annotated as direct regulatory genes of pollen development showed higher abundance in fertile buds. Interestingly, unigene28529 (*BnNZZ/SPL*) was the first unigene that showed a difference in the regulatory network of anther development. Unigene19392 (*BnAG*), which acts as upstream gene of unigene28529, showed no difference in expression [32]. Therefore, it can be presumed that the lack of *Rfp* emancipates the male-sterile gene in mitochondria. Through a series of bioprocesses, this male-sterile gene leads to serial inhibition of the downstream genes by regulating *BnNZZ/SPL*, a pivotal regulator of sporogenesis [33,34]. Consequently, the fertile buds become sterile before the formation of sporogenous cells. It has been reported that nuclear-mitochondrial interaction results in CMS in previous studies [35,36]. These genes were inhibited rather than silenced, which consequently led to the formation of only a few pollen sacs in the sterile buds.

Although *orf224* and *atp6* were both up-regulated in sterile buds, the change in *orf224* expression was twice that of *atp6*, which might have been caused by the truncation of most of the *orf224* transcripts in the presence of *Rfp*[8]. Nevertheless, what has been discussed above indicates that energy supply was much more plentiful in the fertile samples. We speculate that even though the *orf224/atp6* gene was normally transcribed, the translation of it was defective, or the translated protein could not form a functional advanced structure. After all, the existence of integral *orf224* led to the insufficiency of ATPase protein 6, which caused the energy deficiency in the sterile buds. In other studies, the candidate cytoplasmic male sterile genes have also had a close relationship with ATPase genes, such as *atp2*, *atp6*[37,38].

Comparative profiling was performed between the two transcriptomes. It was found that metabolism was still the principal activity in buds. Unigenes that involved in pyrophosphatase and nucleoside-triphosphatase activity were down-regulated, suggesting an energy deficiency in sterile buds. The depauperate bud activity was elucidated by the weakening of transcription and translation, which is similar to the results of an earlier study of *Arabidopsis*[39,40]. Different distributions of energy and materials may strongly influence fertility, and the regulation of sterility is very complicated. At the transcriptional level, mRNA plays an important role; besides that, small RNA also participates in the process and makes the regulation network more complete [41,42].

**Figure 8:**
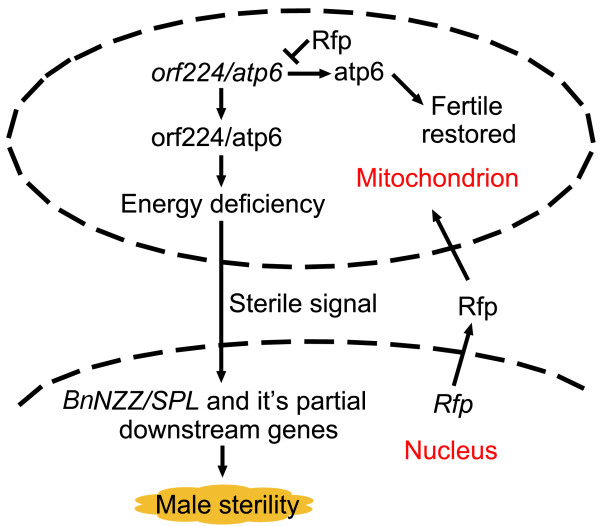
**Proposed model for sterile and restored mechanism of *pol *** **CMS.**

We hypothesized that *orf224* was integrally transcribed because of the lack of the *Rfp* gene in *pol* CMS and that this led to malformation of the advanced structure of atp6, which subsequently caused an energy deficiency. Therefore, the exchange of materials between the nucleus and cytoplasm was blocked. The expression of some genes that were located in the nucleus and function in pollen development was inhibited, and *BnNZZ/SPL* was the first inhibited gene in temporal order. Finally, anthers could not differentiate sporogenous cells and became sterile in *pol* CMS (Figure 8).

## Conclusions

In this study, the different bioprocesses and genes that regulate pollen development were analyzed through a high-throughput sequencing approach. Combined with the investigation of the *orf224/atp6* gene region, we propose a speculative model for the sterility and restoration mechanism of *pol* CMS. These results will promote the study of sterility mechanisms in more detail and the cloning of restorer genes. Additionally, the assembled *B. napus* unigenes obtained in this study will contribute to other transcriptomic and genomic studies.

## Methods

### Plant materials and RNA preparation

Fertile and sterile flower buds of *pol* CMS in *B. napus* were used in this study*.* They were near-isogenic lines (NIL) and were both cultivated in the same experimental plot in Huazhong Agriculture University (Wuhan, Hubei Province, China). Buds with a length of 0–1 mm were stripped from three different plants for transcriptomic profiling. All fertile buds were gathered together (as were sterile buds), snap-frozen in liquid nitrogen and kept at -80°C for further use. Total RNA was isolated according to the instructions of the TRIzol kit (Invitrogen, USA) and purified using a mRNA purification kit (Promega, China) following the manufacturer’s protocol.

### cDNA library construction and RNA-Seq

The mRNA was reverse transcribed by Powderscript^TM^ II (Takara, China), and then double-stranded cDNA was amplified according to the user manual of the SMART^TM^ cDNA construction kit. Finally, double-stranded cDNA was purified using a DNA purification kit (QIAGEN, Germany) to generate high quality cDNA. Approximately 10 μg of sheared cDNA was then prepared for Illumina sequencing according to the manufacturer’s protocols. Libraries were prepared from a 300–500 bp size-selected fraction following adapter ligation and agarose gel separation. The libraries were sequenced using a paired-end read protocol with 100 bp of data collected per run on the Illumina Hiseq 2000.

### Analysis of Illumina sequencing results

Clean reads were obtained from raw data by filtering the adaptor sequences and the low-quality sequences using SolexaQA [43]. Then, the pair-end clean reads were *de novo* assembled into contigs by Trinity. After that, TGI Clustering tools were used to obtain the non-redundant unigenes [44].

Functional annotation of unigenes was performed using BLASTX searches against the NR, Swiss-Prot and COG databases (E-value < 1e-5). The GO annotations for the unigenes were determined using Blast2GO [31]. Then, the results were submitted to WEGO to obtain the GO classification graph. The raw reads were deposited in the NCBI SRA (Short Read Archive) with the accession number SRA069852. The assembled unigenes are shown in Additional file 4.

### Mapping and expression level analysis

The reads of the fertile and sterile samples were mapped back to our *de novo* assembling results separately using Tophat [45]. Then, the differentially expressed unigenes were obtained using Cufflinks [46]. The results were submitted to KAAS and Blast2GO for further interpretation.

### Real-time quantitative PCR (qRT-PCR) verification

First-strand cDNA was synthesized using the RevertAid First Strand cDNA Synthesis Kit (Thermo, USA), from 1,200 ng total RNA. Gene-specific primers were designed based on the selected unigene sequences (Additional file 5). Reactions were performed with the SYBR Green Realtime PCR Master Mix (TOYOBO, Japan) in a Bio-Rad CFX96 instrument. Three biological replicates for each sample and three technical replicates were performed and the relative expression level was calculated using the 2^-ΔΔCt^ method. The actin gene was used to normalize the gene expression.

### Semi-thin sections and light microscopy

Sterile and fertile flower buds were fixed and embedded in resin using Technovit Embedding Kits (Germany). Then, semi-thin (2.5 μm) sections were obtained using an automatic microtome (Microm HM 360, Thermo). The sections were stained with 0.1% toluidine blue O for 30–60 sec at room temperature and observed with a Nikon Eclipse 80i microscope (Nikon, Japan). Images of the anthers in different stages were captured with a Nikon DS-Ri1 camera (Nikon, Japan).

### Availability of supporting data

The project was submitted to NCBI BioProject with BioProject ID: PRJNA193477 (http://www.ncbi.nlm.nih.gov/bioproject/?term=PRJNA193477). The raw reads were deposited in NCBI SRA (Short Read Archive) with the accession number SRA069852 (http://www.ncbi.nlm.nih.gov/sra/?term=SRA069852). The assembled unigenes were deposited in the Additional file 4: *De novo* assembled transcripts sequences. The DGEs and their annotations were deposited in the Additional file 3: Expressions and annotations of the differentially expressed unigenes.

## Competing interests

The authors declare that they have no competing interests.

## Authors’ contributions

BY conceived of the study. HA carried out the data analysis and molecular woks. ZY obtained the pol near-isogenic line (NIL) and prepared the RNA. HA and ZY executed anatomical work. HA and BY drafted the manuscript. JW, JS, JT, CM and TF provided suggestions, funds and experimental conditions. All authors have read and approved the final manuscript.

## Supplementary Material

Additional file 1 Summary of sequencing results. Click here for file

Additional file 2 **19 genes related to pollen development.** *: detected or confirmed by qRT-PCR. Click here for file

Additional file 3 **Expressions and annotations of the differentially expressed unigenes.** *: detected or confirmed by qRT-PCR. Click here for file

Additional file 4 ***De novo *** **assembled transcripts sequences.** Click here for file

Additional file 5 Primer sequences for qRT-PCR. Click here for file
